# Systematic large-scale meta-analysis identifies a panel of two mRNAs as blood biomarkers for colorectal cancer detection

**DOI:** 10.18632/oncotarget.8108

**Published:** 2016-03-16

**Authors:** Maria Teresa Rodia, Giampaolo Ugolini, Gabriella Mattei, Isacco Montroni, Davide Zattoni, Federico Ghignone, Giacomo Veronese, Giorgia Marisi, Mattia Lauriola, Pierluigi Strippoli, Rossella Solmi

**Affiliations:** ^1^ Department of Experimental, Diagnostic and Specialty Medicine (DIMES), Unit of Histology, Embryology and Applied Biology, University of Bologna, Bologna, Italy; ^2^ Centre of Molecular Genetics, “CARISBO Foundation”, Bologna, Italy; ^3^ Department of Medical and Surgical Sciences (DIMEC), University of Bologna, Bologna, Italy; ^4^ Biosciences Laboratory, Istituto Scientifico Romagnolo per lo Studio e la Cura dei Tumori (IRST) IRCSS, Meldola, Italy; ^5^ Interdepartmental Center for Cancer Research “Giorgio Prodi” (CIRC), S. Orsola-Malpighi Hospital, University of Bologna, Bologna, Italy

**Keywords:** TRAM: Transcriptome Mapper, CRC: colorectal cancer, CTC: circulating tumour cells

## Abstract

Colorectal cancer (CRC) is the third most common cancer in the world. A significant survival rate is achieved if it is detected at an early stage. A whole blood screening test, without any attempt to isolate blood fractions, could be an important tool to improve early detection of colorectal cancer. We searched for candidate markers with a novel approach based on the Transcriptome Mapper (TRAM), aimed at identifying specific RNAs with the highest differential expression ratio between colorectal cancer tissue and normal blood samples. This tool permits a large-scale systematic meta-analysis of all available data obtained by microarray experiments. The targeting of RNA took into consideration that tumour phenotypic variation is associated with changes in the mRNA levels of genes regulating or affecting this variation.

A real time quantitative reverse transcription polymerase chain reaction (qRT- PCR) was applied to the validation of candidate markers in the blood of 67 patients and 67 healthy controls. The expression of genes: *TSPAN8, LGALS4, COL1A2* and *CEACAM6* resulted as being statistically different.

In particular ROC curves attested for *TSPAN8* an AUC of 0.751 with a sensitivity of 83.6% and a specificity of 58.2% at a cut off of 10.85, while the panel of the two best genes showed an AUC of 0.861 and a sensitivity of 92.5% with a specificity of 67.2%.

Our preliminary study on a total of 134 subjects showed promising results for a blood screening test to be validated in a larger cohort with the staging stratification and in patients with other gastrointestinal diseases.

## BACKGROUND

Colorectal cancer (CRC) is the third most common cancer in the world, with nearly 1.4 million new cases detected in 2012. A significant survival rate is achieved if the primary tumour is detected at an early stage [1–3].

Most CRC develops in a multistep process, starting with benign precancerous adenomas, which develop into aggressive metastatic carcinoma [4]. This makes early detection crucial to benefit the chances of a positive outcome for CRC patients [5].

Multiple non-invasive screening modalities have been investigated including faecal tests that detect the presence of haemoglobin or blood in the stools [5–7], and improved faecal test methods that add an integral DNA extraction [8]. Very recently, Imperiale at al. [9] proposed a multi-target stool DNA test, Cologuard (Exact Sciences Corporation, Madison WI), approved by the American Food and Drug Administration (FDA), but further adjustments are necessary because of the high rate of false positive stool DNA results [10]. Moreover, in 2010, the CellSearchVR system (Veridex, Johnson-Johnson, USA) for circulating tumour cell (CTC) enumeration in metastatic colorectal cancer (mCRC), based on immunofluorescent detection, received FDA approval [11, 12]. Recently, a combinatorial panel of seven mRNA biomarkers in blood: annexin A3 (*ANXA3*), C-Type Lectin Domain Family 4, Member D (*CLEC4D*), Lamin B1 (*LMNB1*), Proline Rich Gla (G-Carboxyglutamic Acid) 4 (Transmembrane) (*PRRG4*), Tumor Necrosis Factor, Alpha-Induced Protein 6 (*TNFAIP6*), Vanin 1 (*VNN1)* and Interleukin 2 Receptor, Beta (*IL2RB*) has been proposed by Marshall et al. [13] (ColonSentry^®^, Canada - Enzo Biochem. USA). The test has recently been approved by the New York State Department of Health as a test to determine a person's risk of having CRC [14]. The search for markers as a screening tool in the patient's blood represents an active field of research for early detection of colorectal cancer. Numerous reports include coding mRNAs, microRNAs (miRNAs), protein, metabolic, DNA mutation [15] and methylation markers. Currently, the principal trends of research into mRNA candidate markers generally involve several types of experimental evidence: circulating tumour cells (CTCs) [13, 16], cancer stem cells (CSCs) [17] and circulating free RNA (cfRNA) [18]. The metastatic spread occurs very early in the tumour's development; hence specific and sensitive detection of CTCs has become crucial for diagnosis [19]. A quantitative polymerase chain reaction (qPCR) has recently been described as a good CTC quantification method [19–22].

The cfRNAs could be good blood cancer biomarkers, as they may be more informative, specific and accurate than protein biomarkers [23–26]. Various research groups have investigated the potential use of circulating mRNA as markers for cancer. The general experimental strategy is to employ microarray technology for mRNA expression profiling, which is followed by a real time quantitative reverse transcription polymerase chain reaction (qRT-PCR) validation. The specimens used are either mRNA extracted directly from blood, serum/plasma or from isolated blood cells [6].

Our research, as well as that of other groups, argues that a test on the whole blood, without any attempt to isolate CTCs or CSCs or any blood fraction, is much simpler to conduct (in the perspective of a wide use in oncology practice), and is not affected by loss of CTCs or CSCs, whose number is critical for the success of the test, during purification [27]. The targeting of RNA makes use of the fact that tumour phenotypic variation is associated with changes in the mRNA levels of genes regulating or affecting this variation. This has led to the widespread use of a qRT-PCR assay in clinical diagnostics [28]. RT-PCR offers several advantages over other detection methods in terms of high sensitivity and specificity. To date, the main issue has been to identify a biologically suitable mRNA with a clear cut-off of its expression values between CRC and healthy subjects. We have previously addressed this question by exploiting pre-built online databases of differential expression among tissues or by performing ad hoc microarray experiments, which compare cancer patients' blood with healthy reference subjects [29–33]. However, to our knowledge, no study has to date been designed which undertakes a comprehensive, systematic analysis of gene expression in CRC and blood while addressing three major problematic issues: including the largest possible number of available gene expression profile datasets from different sources, integrating the data at level of each transcribed known or uncharacterized locus by uniform gene name assignment, and performing robust intra-sample and inter-sample normalization.

We used a novel approach based on the Transcriptome Mapper (TRAM) [34–36] tool, aimed at identifying specific RNAs [37] with the highest differential expression ratio between CRC and normal blood samples, and therefore possibly suitable for detection of CRC-related changes in patients' blood. TRAM is based on a systematic large-scale meta-analysis of all available data obtained by microarray experiments.

The best candidate RNAs identified by our computational biology approach out of 38,104 human transcripts whose expression level was compared between CRC (*n* = 349) and blood (*n* = 200) samples, were tested by a quantitative RT-PCR analysis. A group of 134 healthy volunteers and patients with diagnosed CRC at various stages was used.

## RESULTS

### TRAM based data set meta-analysis

A systematic meta-analysis of differential gene expression in CRC and normal blood was conducted to identify the mRNAs with the highest expression ratio between CRC and blood, in order to select the best candidate biomarkers. The Transcriptome Mapper (TRAM) tool allowed the management of experimental platforms with different numbers of genes in order to maximize information that could be extracted from the dataset [34]. Following a comprehensive search, a total of 37 GEO Series were selected for CRC along with an additional 23 series for blood. The selected series included a total of 2,532 and 958 samples, respectively. Random sampling was done to further select the first 10 listed samples for each series including more than 10 samples, thereby reducing the number of samples analysed to 349 for CRC and 200 for blood (14% and 21% of the total, respectively; Supplementary Table 1). Analysis of the final resulting integrated and normalized dataset allowed the identification of genes, among a total of 38,104 expressed loci with an available expression value in both ‘A’ (CRC) and ‘B’ (blood) pools, with the absolute greatest ‘A/B’ ratio between expression value in CRC tissue vs blood cells. The list (Supplementary Table 2) comprises CRC overexpressed loci with a ratio greater than 32:1, which could be considered as a threshold for the choice of the best candidate RNAs. In this case, a clearly identifiable difference equivalent to 5 cycles of PCR, in theory, is expected between cases and controls during the exponential phase of amplification.

### CRC marker selection

A further screening of the complete TRAM database results (Supplementary Table 2) was carried out, in order to search for loci whose mean expression value came from a number of data points greater than 50% of the sample number for each pool, i.e. > 175 for CRC and > 100 for blood. In this way, transcripts whose mean expression value was not assessed in a large fraction of the sample pool were excluded from the experimental validation. This was often the case of uncharacterized UniGene expressed sequence clusters coded by the “Hs.” prefix. This filtering led to the selection of the 15 best theoretical candidates (Table 1).

**Table 1:** Selected candidate markers (the first 15 loci with the highest ‘A/B’ ratio and with a number of data points greater than 50% of the sample number for each pool)

Gene name	Value ‘A’*Colorectal cancer*	Value ‘B’*Normal blood*	Ratio‘A/B’	Location	Data points ‘A’	Data points‘B’	SD as % of expression ‘A’	SD as % of expression ‘B’
***TSPAN8***	2,313.03	13.12	176.36	chr12	349	185	67.66	424.15
***EPCAM***	2,111.87	13.60	155.27	chr2	354	222	69.86	82.81
***SPINK1***	1,086.88	12.68	85.70	chr5	368	215	99.51	107.87
COL3A1	862.27	10.10	85.35	chr2	1291	527	140.54	110.39
CEACAM5	2,074.89	24.44	84.88	chr19	572	315	132.13	144.57
***COL1A2***	989.85	12.79	77.42	chr7	767	549	115.82	131.00
***CDH1***	825.52	11.87	69.56	chr16	573	455	120.84	105.73
***LGALS4***	1,980.67	29.50	67.15	chr19	369	185	77.43	104.96
KRT18	1,719.60	25.69	66.93	chr12	482	318	88.43	111.69
SLC26A3	800.45	12.31	65.01	chr7	369	195	187.48	138.12
REG1A	776.24	12.54	61.91	chr2	346	185	191.25	75.25
FN1	664.80	11.22	59.25	chr2	1588	902	144.43	125.93
LUM	556.84	9.40	59.22	chr12	403	262	108.62	83.50
***CEACAM6***	2,245.12	38.49	58.33	chr19	583	274	65.15	480.79
KRT20	731.52	12.58	58.13	chr17	372	182	119.91	85.52

Colorectal cancer tissue (‘A’), normal blood (‘B’).

‘Value’: mean gene expression value normalized across all the pool samples; ‘Data points’: number of spots with an expression value for the locus; ‘SD’: standard deviation for the expression value expressed as a percentage of the mean. Gene name in bold: selected for experimental validation.

In order to select for transcripts with absolute high abundance, considering that the expression value is expressed as a percentage of the mean value in the integrated expression profile (i.e., 1,000 = ten times the mean), we further excluded the last loci of the list (from *SLC26A3* to *KRT20*) except *CEACAM6*, which has a high absolute expression value. We then excluded carcinoembryonic antigen-related cell adhesion molecule 5 (*CEACAM5*), collagen, type III, alpha 1 (*COL3A1*), and Keratin 18 (*KRT18*) because of the existence of pseudogenes, which not allow mRNA specific PCR primer design, to distinguish between mRNA and DNA contamination. Hence the seven final candidate markers left for further evaluation were: Tetraspanin 8 (*TSPAN8*), Epithelial Cell Adhesion Molecule (*EPCAM*), Serine Peptidase Inhibitor, Kazal Type 1 (*SPINK1*), Collagen, Type I, Alpha 2 (*COL1A2*), Cadherin 1, Type 1, E-Cadherin (Epithelial) (*CDH1*), Lectin, Galactoside-Binding, Soluble, 4 (*LGALS4*), and Carcinoembryonic Antigen-Related Cell Adhesion Molecule 6 (Non-Specific Cross Reacting Antigen) (*CEACAM6*) (Table 1).

### Quantitative analysis of blood mRNA markers

Each RNA sample (patients or healthy) was tested for quality and the expression of the listed candidate markers was detected by quantitative PCR and normalized on the housekeeping gene *B2M* as reported by Hamm et al. [38]. In the same data sets (CRC and blood tissues), which we employed in our meta-analysis (Supplementary Table 2), *B2M* displayed the lowest variability between CRC and normal groups (calculated as Standard Deviation compared to the Mean), being the most stably expressed gene in comparison with other most commonly used reference housekeeping genes such as *ACTB* and *GAPDH*.

The standard curve of each primers pair, along with the slope values, is shown in Supplementary Figure 1 and Supplementary Table 3.

The tested genes showed a unique peak in melting curve analysis and none of the negative controls gave detectable amplification values, corroborating the specific amplification.

The normalized mRNA expression levels in blood, indicated as Delta CT (Threshold Cycle) were reported in Table 2. Following a first round of analysis testing 39 CRC and 36 normal samples, the four best identified markers were tested in the complete sample set (Table 2). The relative expression level of the candidate markers appears to be extremely specific for these genes. The expression of four of the seven analysed genes: *TSPAN8, LGALS4, COL1A2* and *CEACAM6*, displayed statistically significant differences between cases and controls.

**Table 2:** mRNAs expression levels of the indicated markers

	CRC patients number (*n*)	CRC patientsMean ΔCt ± SD	Controls number	ControlsMean ΔCt ±SD	*p*-value	AUC	Sensitivity%	Specificity%
*TSPAN8*	67	9.41 ± 2.00	67	11.33 ± 1.72	0.00000002	0.751	83.6	58.2
*EPCAM*	39	11.23 ± 1.36	36	11.83 ± 1.23	0.08	0.631	-	-
*SPINK 1*	39	11.88 ± 2.87	36	11.85 ± 2.59	0.9	0.503	-	-
*COL1A2*	67	9.59 ± 2.14	67	11.45 ± 1.92	0.0000005	0.718	73.1	59.7
*CDH1*	39	9.9 ± 0.9	36	9.5 ± 1.08	0.2	0.581	-	-
*LGALS4*	67	14.43 ± 1.28	67	12.89 ± 1.97	0.0000004	0.746	82.1	61.2
*CEACAM6*	67	13.23 ± 1.24	67	12.34 ± 1.89	0.0017	0.632	65.7	61.2
*Panel: TSPAN8 + LGALS4*	67	-	67	-		0.861	92.54	67.16

Statistical values between CRC patients and controls, AUC from ROC curves, sensitivities, specificities are reported

‘CRC’: colorectal cancer; ‘ΔCt'': delta cycle threshold; ‘Ct’: threshold cycle; ‘SD’: standard deviation; ‘AUC’: area under the ROC curve. *p*-value: 2-way Anova.

### Diagnostic value of blood mRNA markers for CRC

In order to evaluate the diagnostic accuracy in term of specificity and sensitivity of the candidate markers, the Receiver Operating Characteristic (ROC) curve analysis was applied. Specifically, the analysis of individual markers was performed by using the MedCalc software. This tool performs statistical test that allows the user to obtain ROC data sensitivity, specificity, and the Youden index (which provides the threshold value that minimizes the probability of finding false positives and false negatives), in order to determine a cutoff for each marker, as reported in Table 2. An initial training/validation test was performed for *TSPAN8*, by blindly dividing the samples cohort into two subgroups: the first one serving as training, the second as validation set (as reported in Supplementary Figure 2C). The test returned a good match for the two subsets, but due to the still relatively small cohort tested, we constructed the further ROC curves, without any stratification of the samples.

**Figure 1:**
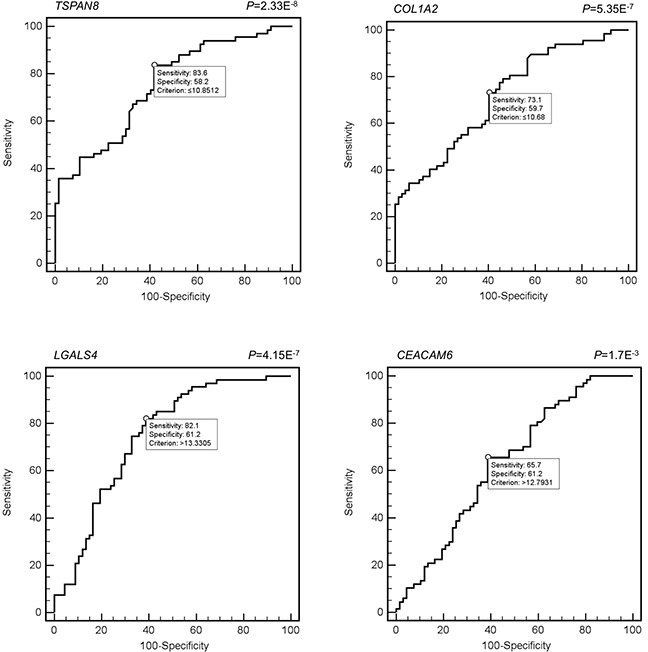
ROC curves of *TSPAN8, LGALS4, COL1A2* and *CEACAM6*

Once the best markers according to ROC analysis has been selected, we integrated the 4 candidate markers values (*TSPAN8, LGALS4*, *COL1A2* and *CEACAM6*), into one variable via Discriminate Analysis by using SPSS. More specifically, the SPSS tool calculated the discriminating power of the simultaneous use of the best scores obtained with MedCalc. Hence, we built a model of multiple logistic (binary logistic regression). In this model the dependent variable is the presence/absence of the disease (normal Vs cancer) and the independent variable is the circulating level of markers used to construct the ROC curve, creating a graph of probability. Through this analysis, we examined whether using a panel of biomarkers as opposed to using them individually improves their discriminating power. The highest diagnostic accuracies closest to 1 were found for *TSPAN8* (AUC 0.751), *LGALS4* (AUC 0.746), *COL1A2* (AUC 0.718) and *CEACAM6* (AUC 0.632). The corresponding graphical elaboration for each marker is reported in Figure 1.

### Assessing diagnostic potential of the mRNA panel

**Figure 2:**
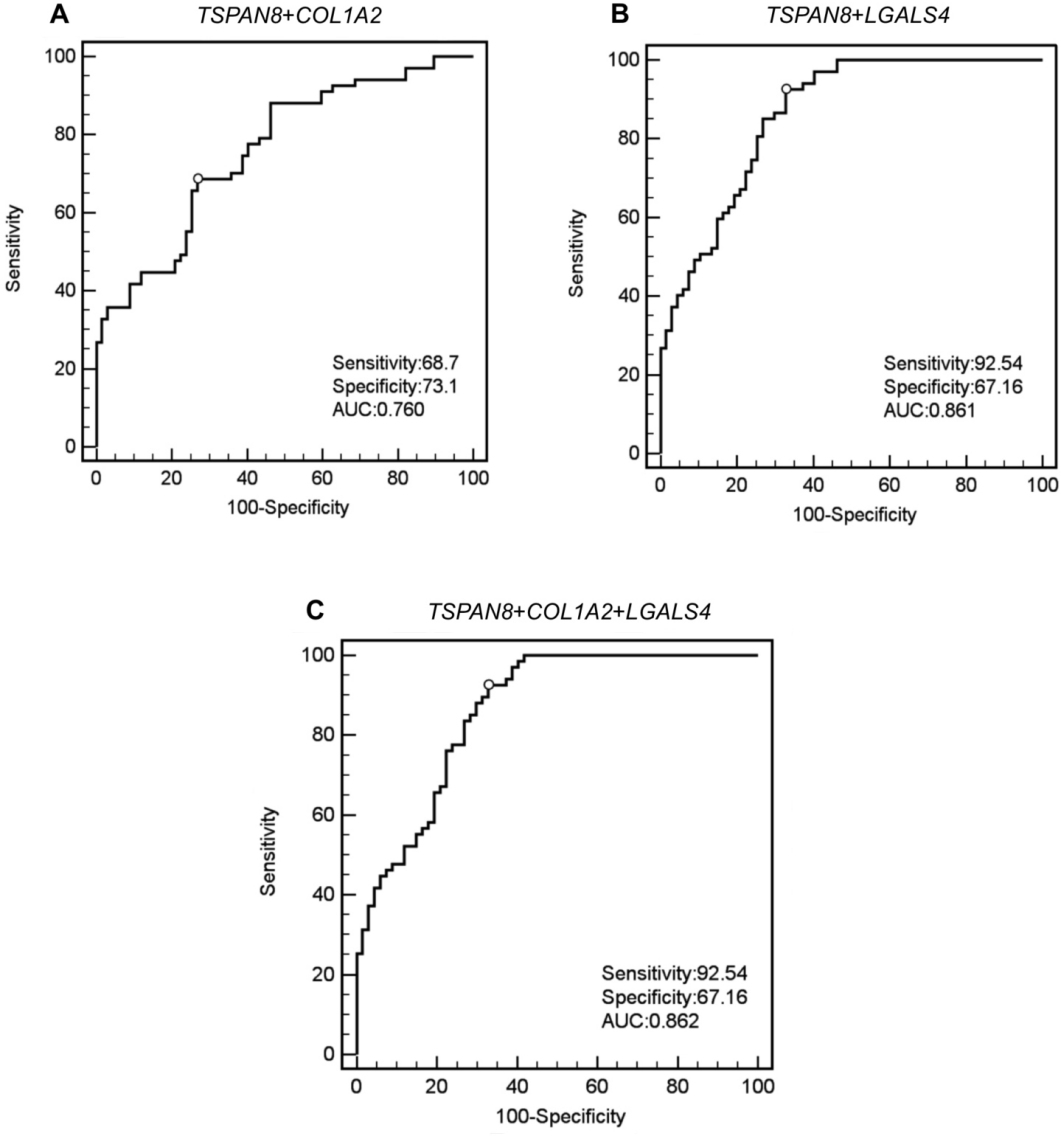
ROC curves of panels of the indicated marker gene combinations (**A**) *TSPAN8* + *COL1A2*; (**B**) *TSPAN8* + *LGALS4*; (**C**) *TSPAN8*+*COL1A2*+*LGALS4*.

To assess the potential use of the selected mRNA candidates as a diagnostic panel for CRC, ROC analysis was performed on the validated data set for every possible combination. *TSPAN8* and *COL1A2* combination displayed 68.7% sensitivity and 73.1% specificity (Figure 2A). The combination of *LGALS4* and *TSPAN8* displayed 67.16% specificity and 92.54% sensitivity (Figure 2B), with a positive predicted value (ppv) of 76.25% and negative predicted values (npv) of 88.25%, as reported in Supplementary Table 4. For the single genes, specificity reached about 61% and 58.2% respectively, while sensitivity measured 82.1% and 83.6% respectively. The combination of *TSPAN8* with *LGALS4* and *COL1A2* returned the same sensitivity and specificity as *TSPAN8* and *LGALS4* combination (Figure 2C).

## DISCUSSION

A whole blood screening test could be an important addition to improving CRC screening and early cancer detection. We searched for the candidate markers with the TRAM software application, which executed the basic computational biology tasks needed for this study. TRAM is a powerful bioinformatics tool, which allowed the complete comparison of differential gene expression between colon cancer and normal blood tissues without any *a priori* knowledge. It also allowed us to parse, integrate and analyse gene expression data relative to all the described human RNAs. Notably the bioinformatics analysis blindly detected some genes that are already known as markers in solid carcinomas. Furthermore, by using the whole blood as a detection tool, without any attempt to fractionate, we were able to test both mRNA molecules and circulating free RNAs. The latter molecules have been described in the blood of CRC patients as reflecting the circulating tumour burden [23]. These molecules are stable in the bloodstream with a variable half-life ranging from 15 minutes to several hours [37, 39–40]. In our study, we assumed to detect any RNA molecules of either cfRNA or CTCs origin. Under these conditions our analysis confirmed four out of the seven candidate markers as potential CRC blood markers. The expression of these genes: *TSPAN8*, *LGALS4*, *COL1A2* and *CEACAM6* in the whole blood may be useful in the detection of CRC.

The combination of *TSPAN8* and *LGALS4* shows promising values of sensitivity (92.5%) and specificity (67.16%), competing with the widely used faecal occult blood test (FOBT), or the faecal immunochemical test (FIT) (74% and 95%) and Cologuard (92% and 87%) [8, 41]. To our knowledge, this is the first report examining *TSPAN8* mRNA expression in the blood for CRC diagnosis. Little is known about TSPAN8 or Co-029 protein in cancer. Previous studies described this protein as an invasive marker that enables melanoma cells to cross the basement membrane, leading to dermal invasion and progression to metastasis. Hence TSPAN8 was suggested a promising target in early detection, at least in melanoma [42].

The others markers screened include cell surface glicoproteins such as *CEACAM6* [43–44] and stromal genes such as *COL1A2* and *LGALS4*. The latter regulates cell motility on collagen I by cooperating with the E-cadherin/p120-catenin (p120ctn) complex. COL1A2, the most abundant protein in the human body, indeed increases the synthesis in colon malignancy and reveals a considerably higher expression in stage II tumours, suggesting that its activation is an early event in CRC tumorigenesis [45, 46]. In our study, *TSPAN8* as well as *COL1A2* expression is significantly lower in the blood of healthy subjects compared to patients. On the contrary, the expression level of the two other candidate markers *LGALS4* and *CEACAM6* display opposite trends, with higher levels in normal healthy blood compared to cancer blood. These results find support in the literature, which shows, at least for *LGALS4*, associated high expression in the normal small intestine, colon, and rectum, while in colorectal cancers conditions the expression levels fall. Hence it was suggested that it functions as a tumour suppressor by inhibiting cell proliferation. Furthermore, decreased *LGALS4* mRNA levels may be an early event in colon carcinogenesis [47]. We argue that products whose expression in colon cancer is very different from that in normal mucosa are potential biological markers of the progression of malignant lesions [48].

Interestingly, by combining *TSPAN8* and *LGALS4*, which display specular expression in cancer and/or normal blood (*TSPAN8* is higher in CRC blood, whereas *LGALS4* is lower), we detected promising values of sensitivity and specificity compared to the markers alone. The *TSPAN8* and *COL1A2* combination displays an improvement in specificity increasing to 73.1%.

Undoubtedly, our results require extending the study to a larger cohort, which might also allow the staging stratification and the inclusion of patients with other gastrointestinal diseases. It would also be interesting to verify whether the staging and tumour invasiveness display a different amount of the panel markers. So far our results suggest that a panel, reflecting the heterogeneity of the disease, is more successful at diagnosing CRC than a single biomarker [7, 49]. Both stromal genes and cancer membrane glycoprotein appeared to be useful contributions to the cancer gene expression profile. Indeed, interactions between the stroma and parenchyma of tumours are increasingly recognized as important factors in tumour biology and clinical outcome [50, 51].

In summary, our preliminary study showed that the *TSPAN8, LGALS4* and *COL1A2* mRNA expression in blood is a reliable tool for detecting the presence of CRC, considering the levels of sensitivity and specificity evidenced. Further studies will be needed to test the panel of biomarkers in the screening setting, where the detection of early cancers or precancerous lesions is a crucial target. Both preneoplastic lesions and high-risk adenoma patients should be questioned in future studies, in order to test these markers in the context of early detection.

## MATERIALS AND METHODS

### Database search and selection

The Gene Expression Omnibus (GEO) functional genomics repository was searched for: “Colorectal cancer AND Homo sapiens [ORGANISM]”. The terms “Colon cancer” and “Rectal cancer” were also used. In addition, the search for “Whole blood” [Sample Source] AND Homo sapiens [ORGANISM]” was used to retrieve datasets in the same database, thereby generating a pool serving as a comparison set to highlight CRC-specific differential gene expression compared to blood cells. The searches were carried out until February 2012. Search results were then filtered using inclusion and exclusion criteria as explained below.

The inclusion criteria of datasets in the analysis were: experiments of the type “Expression profiling by microarray”; primary tissue; any age or sex of the subject; cancer of any stage (for CRC); unfractioned peripheral blood; unstimulated blood cells; availability of the raw or pre-processed data for the single channel of the tissue of interest in the case of two-channel experiments.

Exclusion criteria were: cell line samples; non malignant adenoma tissues; metastatic cells; patients on treatment; cord blood; blood fractions, e.g. peripheral blood mononuclear cells or blood samples subjected to globin mRNA reduction; blood from treated or non-healthy or paediatric subjects; exon arrays (hampering data elaboration by TRAM due to an exceedingly high number of data rows) or platforms using probes split into several distinct arrays for each sample (hampering intra-sample normalization); platforms assaying an atypical number of genes (i.e. < 5.000 or > 60.000).

In order to obtain a quantitative transcriptome map, values from each dataset were linearized when provided as logarithms.

Finally, due to the timing of the TRAM elaboration process, for each series of GEO samples we randomly selected the first 10 listed samples when the sample number was greater than 10. This was done for a total of 349 CRC samples out of 2,532 found and 200 samples of normal blood out of 958 found.

The 349 CRC subjects considered for our meta-analysis were randomly selected from 37 studies performed in different countries (Australia, China, Denmark, Finland, Germany, Hungary, Italy, Japan, Norway, Poland, Singapore, South Korea, Spain, USA). Their clinical data are not completely available for all subjects, and the age range of patients was between 42 and 88 years old. Males and females were equally represented, all colon sites and Dukes stages were included. The 200 blood samples considered were obtained from 23 studies, which were also from different countries (Australia, China, Denmark, Germany, India, Italy, Netherlands, Norway, United Kingdom, USA), and came from healthy subjects with no clinical history of neoplastic disease.

### TRAM (Transcriptome Mapper) analysis

TRAM (Transcriptome Mapper) software allows the import of gene expression data recorded in the NCBI GEO database in tab-delimited text format. It also allows the integration of all data by decoding probe set identifiers to gene symbols via UniGene data parsing, normalizing data from multiple platforms using intra-sample and inter-sample normalization (scaled quantile normalization) [36].

We created a directory (folder) for each tissue, containing all the sample datasets related to the same source (pool ‘A’, CRC, *n* = 349; pool ‘B’, blood, *n* = 200) and selected for the study. The comparisons allowed us to analyse the differential transcriptome maps ‘A’ vs ‘B’, using the ratio of the mean expression values for each locus.

We used an updated version of TRAM, including enhanced resolution of gene identifiers and updated UniGene and Entrez Gene databases (TRAM 1.1, June 2013, freely available at http://apollo11.isto.unibo.it/software), instead of the original 2011 version. The data for 10 platforms used by some of the experiments selected but not provided by the default configuration of TRAM 1.1 (version for “Human”) were then uploaded (GPL80, GPL1449, GPL2006, GPL2895, GPL3121, GPL4811, GPL6370, GPL6883, GPL8432, GPL10665). In briefly, gene expression values were assigned to individual loci via UniGene, intra-sample normalized as a percentage of the mean value and inter-sample normalized by scaled quantile.

### Patients

In order to evaluate the proper number of subjects to question, we performed the analysis of statistical power of the study with the G*Power 3 software for Macintosh OS X system. The sample size needed to achieve a power of 0.80 when analysing results by *t*-test (two-tails) has been estimated to be at least 64 healthy control subjects and 64 patients with diagnosed CRC (for alpha (Type I) error = 0.05 and assuming a medium effect size at d = 0.5).

The study was conducted following approval by the ethical committee of Sant'Orsola-Malpighi Hospital, Bologna, and complied with the Ethical Principles for Medical Research Involving Human Subjects of the Helsinki Declaration. All subjects involved were asked for informed written consent before taking part in the study.

A peripheral blood sample (5 mL) was obtained from 67 healthy donors with no clinical history of neoplastic disease and from 67 unrelated patients with a histological confirmed diagnosis of CRC at any stage, before elective surgery (the patients' main clinical data are summarized in Table 3) and without any chemo or radio adjuvant treatments to the surgery. To reduce contamination of samples with epithelial cells from the needle stick, the first 1 mL of blood was discarded. The family history was determined by questioning each volunteer through a questionnaire they were requested to fill in at the time of the blood withdrawal.

**Table 3:** Patients and control group information

		Cases	Controls	*P* value
	age	68 ± 12	65 ± 14	0.40
Gender	male	34	35	0.42
	female	33	32	
Grading	G1	6	-	
	G2	45	-	
	G3	9	-	
	*N.D.A.	7	-	
Position	Cecum	3	-	
	Ascending c.	17	-	
	Transverse c.	3	-	
	Descending c.	15	-	
	Sigmoid c.	10	-	
	Rectum	19	-	
TNM	T1N0	13	-	
	T2N0	15	-	
	T2N1	3	-	
	T3N0	13	-	
	T3N1	8	-	
	T3N2	6	-	
	T4N0	3	-	
	T4N1	1		
	T4N2	2	-	
	*N.D.A.	3	-	
Dukes	A	28	-	
	B	14	-	
	C	19	-	
	*N.D.A.	6	-	
STAGE	1	25	-	
	2 A/B	15	-	
	3 A/B/C	18	-	
	*N.D.A.	9	-	
BMI	≤ 25	26	-	
	>25	32	-	
	*N.D.A.	9	-	
Smoking History	No Smokers	35	-	
	Smokers	8	-	
	Ex Smokers	22	-	
	*N.D.A.	2	-	
Family History	No Familiarity	56	-	
	Familiarity	8	-	
	*N.D.A.	3	-	

* N.D.A. No data available.

### RNA Extraction

The whole blood, drawn into an EDTA tube, was treated for lysis within one hour of being collected by adding TRIzol LS reagent (Invitrogen, Carlsbad, CA, USA) and total RNA was extracted according to the manufacturer's protocol. Total extracted RNA from 1 mL of blood was subjected to standard ethanol precipitation, and the pellet was dissolved in 15 μL RNase-free water to a final concentration of up to 0.5 μg/μL, and stored at −20°C.

The concentration of all RNA samples was quantified by Nanodrop ND-2000 spectrophotomer (Thermo Fisher Scientific, Waltham, MA).

### qRT-PCR

300 ng of RNA was reverse transcribed with the RevertAid First Strand cDNA Synthesis kit (Carlo Erba Reagents, Milan, Italy) and amplified using the EvaGreen system (Bio-Rad, Hercules, CA), according to the manufacturer's instructions. The list of primers (SIGMA ALDRICH, Milan, Italy) for candidate markers and the reference gene is reported in Table 4.

**Table 4:** Primers used for RT-PCR

Gene Symbol	Gene Name	RefSeqRNA GenBank Accession No.	Primer pairs sequence (5′ → 3′)^a^	RT-PCR product size (base pair)
*TSPAN8*	Tetraspanin 8	NM_004616	ctaagtctgatcgcattgtgaacacaattatggcttcctg	103
*EPCAM*	Epithelial cell adhesion molecule	NM_002354	gtatgagaaggctgagataaagcttcaaagatgtcttcgtcc	147
*SPINK1*	Serine peptidase inhibitor, Kazal type 1	NM_003122	tgaaaatcggaaacgccagacgcggtgacctgatgggattt	89
*COL1A2*	Collagen, type I, alpha 2	NM_000089	gtggttactactggattgacctgccagcattgatagtttc	184
*CDH1*	Homo sapiens cadherin 1, type 1, E-cadherin	NM_004360	cagtacaacgacccaacccacacgctgacctctaaggtgg	99
*LGALS4*	Lectin, galactoside-binding, soluble, 4	NM_006149	ttaccctggtcccggacattagcctcccgaaatatggcac	135
*CEACAM6*	Carcinoembryonic antigen-related adhesion molecule 6	NM_00248	cacagtctctggaagtgctccggccagcactccaatcgt	99
*B2M*	Beta-2-microblobulin	NM_004048	tgcctgccgtgtgaaccatgttgcggcatcttcaaacctccatga	97

a Top: forward primer; bottom: reverse primer (for each gene).

Real-time PCR reactions were performed using the CFX96 instrument (Bio-Rad, Hercules, CA), in duplicate, at 95°C for 10 min, followed by 40 cycles of 95°C for 15 sec and 60°C for 1 min, with melting curve analysis. Each qPCR run always included a negative control lacking cDNA template, and a positive control of cDNA derived from the HT-29 cell line, in which the gene of interest is known to be present. Reaction efficiency (E) was calculated from the slope of the standard curve generated from 10-fold serial dilutions of calibrator cDNA, according to the formula: E = [10 (−1/slope)−1] × 100.

### Statistical analysis

The Student's *t*-test was adopted for the comparison of the expression levels analysed between CRC cases and controls. ROC (Receiving Operating Characteristic) curve analysis was used to assess the accuracy with which the parameters diagnosed CRC, in order to discriminate between patients with CRC and controls. Calculation of both the area under the curve and the corresponding 95% confidence intervals was evaluated using MedCalc version 16 for statistical analyses. To determine the cut off of the markers that allows for the best discrimination between the two groups, the discriminant analysis was performed using SPSS statistical software, version 23. The sets of healthy and cancer patients were considered as grouping variable and the four independent markers grouped together as predicted variable for the panel.

## SUPPLEMENTARY MATERIALS FIGURES AND TABLES






