# Supplementary material for: Systematic large-scale meta-analysis identifies a panel of two mRNAs as blood biomarkers for colorectal cancer detection
Source: Oncotarget. 2016 Mar 16;7(21):30295–306. doi: 10.18632/oncotarget.8108 (PMC5058681; doi:10.18632/oncotarget.8108)
Supplement: Supplementary file 1 [file oncotarget-07-30295-s001.pdf]

Systematic large-scale meta-analysis identifies a panel of two mRNAs as blood biomarkers for colorectal cancer detection

Supplementary Materials

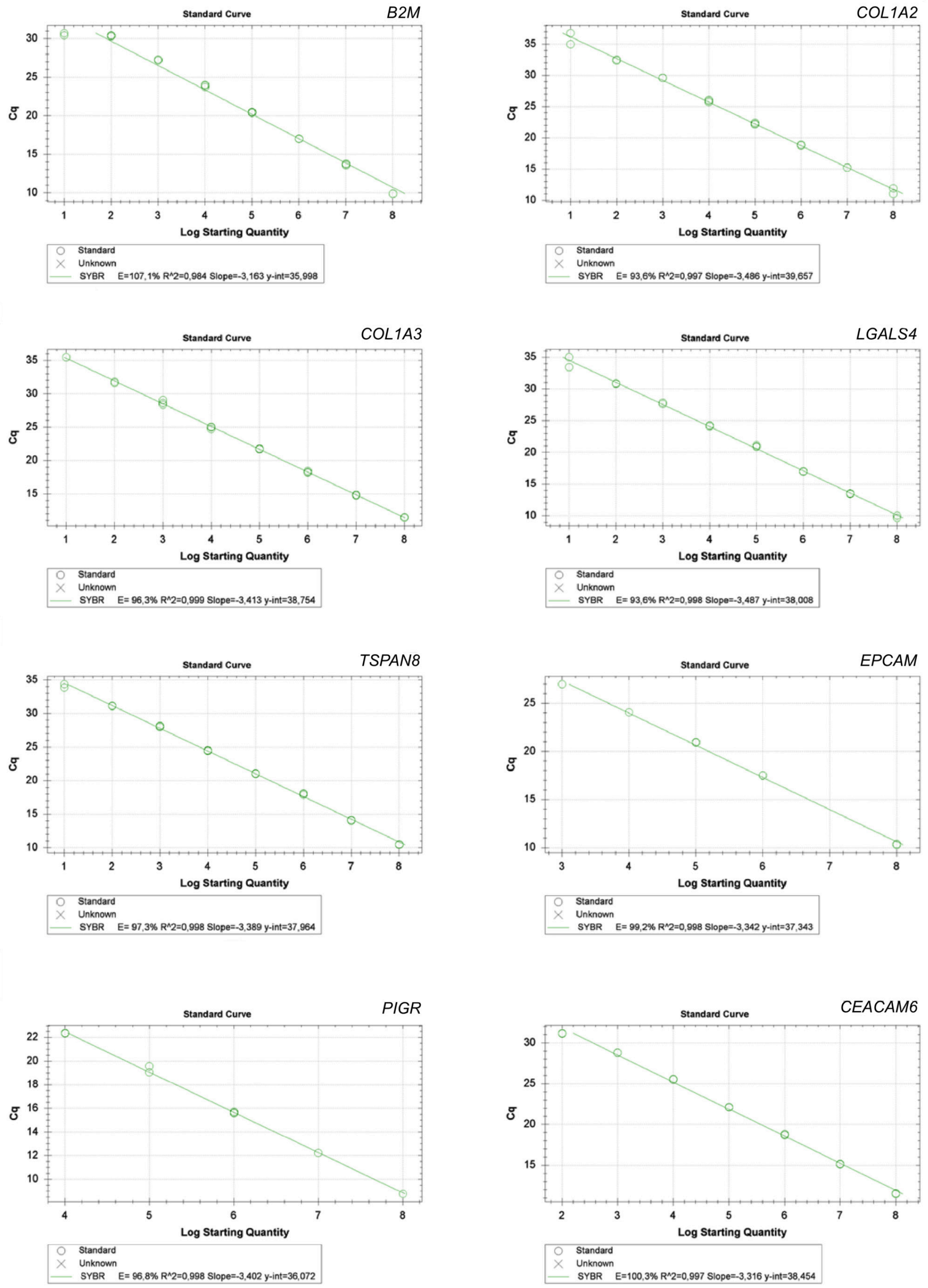

Supplementary Figure S1: Standard curves of primers.

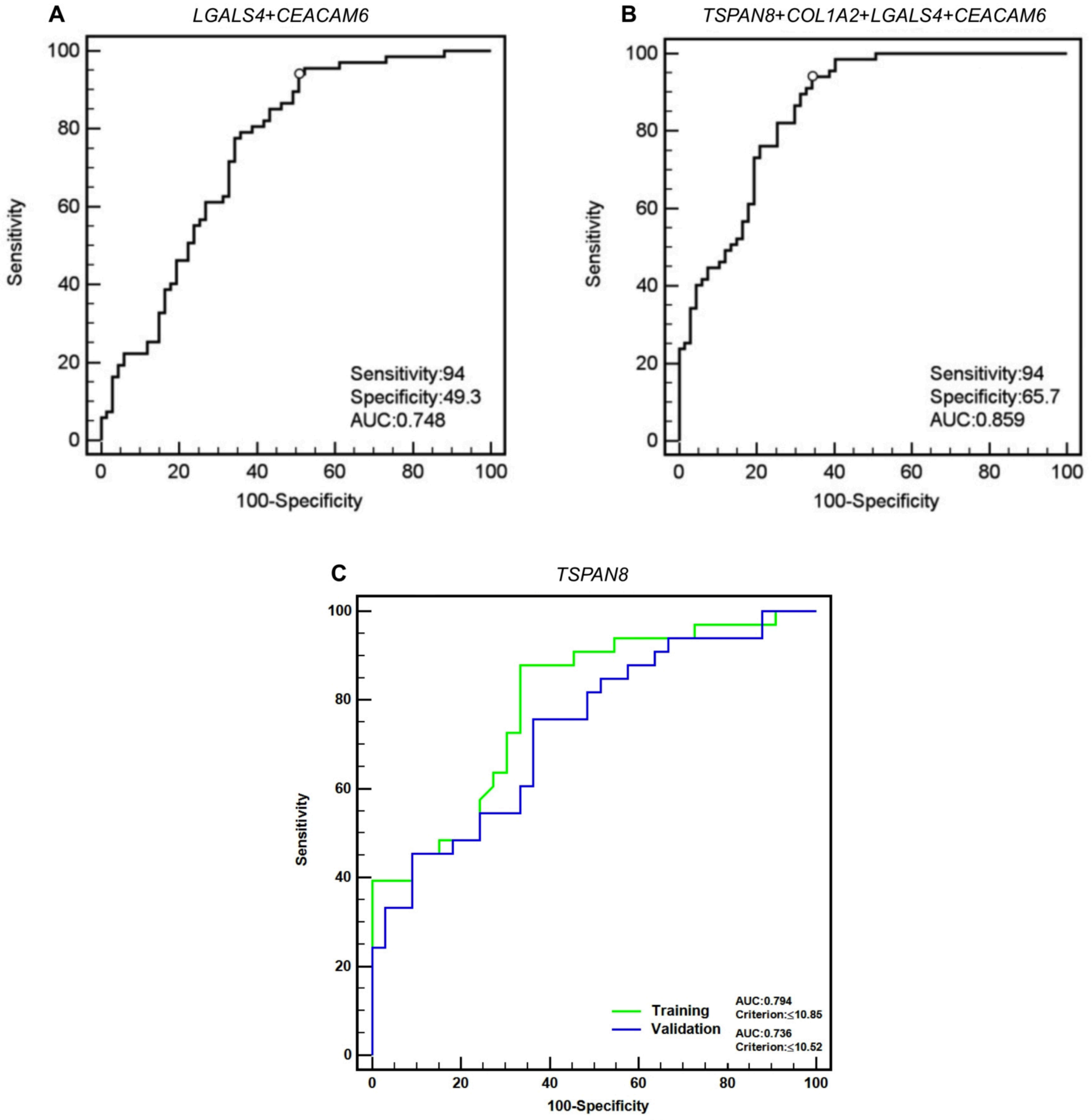

Supplementary Figure S2: ROC curves of panels of the indicated marker combinations. (**A**) *LGALS4 + CEACAM6*; (**B**) *TSPAN8 + COL1A2 + LGALS4 + CEACAM6*; (**C**) Training/validation test for *TSPAN8*.

Supplementary Table S1: Pool 'A' - CRC Samples
Pool 'B' - Blood Samples

Supplementary Table S2: List of 31,804 mapped loci of Transcriptome Map Comparison of CRC vs. Blood Cells (Pool 'A' vs Pool 'B') sorted in descending order of 'A'/'B' expression value. SD = Standard Deviation expressed as percentage of the Expression Value.

Supplementary Table S3: Primers efficiency (%)

	EFFICIENCY (%)	SLOPE
B2M	107.1	−3.163
COL1A2	93.6	−3.486
COL1A3	96.3	−3.413
LGALS4	93.6	−3.487
TSPAN8	97.3	−3.389
CEACAM6	100.3	−3.316
EPCAM	99.2	−3.342
PIGR	96.8	−3.4

Supplementary Table S4: Positive predicted values (PPV) and negative predicted values (NPV) for the indicated markers and the best panel combination.

	PPV	NPV
TSPAN8	67.47%	78.43%
LGALS4	67.90%	77.36%
COL1A2	64.00%	68.97%
CEACAM6	61.64%	63.93%
TSPAN8+LGALS4	76.25%	88.85%
